# TEXAS NURSING HOMES INCREASED DIRECT-CARE STAFFING LEVELS DURING WINTER STORM URI

**DOI:** 10.1093/geroni/igac059.1378

**Published:** 2022-12-20

**Authors:** Dylan Jester, Lindsay Peterson, Ross Andel, David Dosa

**Affiliations:** University of California San Diego, La Jolla, California, United States; University of South Florida, Tampa, Florida, United States; University of South Florida, Tampa, Florida, United States; Brown University, Providence, Rhode Island, United States

## Abstract

Prior work suggests that nursing homes (NHs) increase their registered nurse (RN), licensed practical nurse (LPN), and certified nursing assistant (CNA) staffing levels in anticipation of a major hurricane. However, less is known about NHs’ ability to increase staffing levels during a winter storm. We examined RN, LPN, and CNA staffing levels in 1,170 Texas NHs during Winter Storm Uri from February 13-20, 2021. This storm was characterized by cold temperatures and snow/ice accretion that affected the Texas power grid. Linear mixed effects models were adjusted for profit status, continuing-care retirement community status, resident census, overall star rating, a weighted deficiency score, number of citations for infection control, number of substantiated complaints, and county fixed-effects. After adjustment, Texas NHs decreased RN (β=-0.005;b=-0.002;p=.004) but increased LPN (β=0.022;b=0.009;p<.001) and CNA (β=0.044;b=0.026;p<.001) staffing levels. NHs face unprecedented challenges during winter storms, including maintaining adequate staffing levels to meet the needs of their residents.

